
JOURNAL INFORMATION
==============================
NLM Title Abbreviation: ScientificWorldJournal
Journal ID: ScientificWorldJournal
NLM Journal ID: 101131163
Journal ID: TSWJ

The Scientific World Journal

ISSN: 2356-6140
EISSN: 1537-744X
Publisher: Wiley

ARTICLE INFORMATION
==============================
PMCID: PMC6084381
PMID: 30147565
DOI: 10.1100/tsw.2001.205
Article ID: 574658
Article version: 1
Subjects: Short Report

The Role of XCHK1 in the Inhibition of Apoptosis

Carter Ayesha D.
Sible Jill C.
Publication date: 2001
Electronic publication date: 2001 Oct 11
Volume: 1
First page: 92
Last page: 92
Copyright: Copyright © 2001 Ayesha D. Carter and Jill C. Sible.
Copyright year: 2001
License: This is an open access article distributed under the Creative Commons Attribution License, which permits unrestricted use, distribution, and reproduction in any medium, provided the original work is properly cited.
License URL: https://creativecommons.org/licenses/by/3.0/

Funding: National Institutes of Health GM59688-01A1

==============================
Miami *Nature Biotechnology* Short Reports
*TheScientificWorld* (2001) 1 (S3), 92SR
ISSN 1532-2246; DOI 10.1100/tsw.2001.205

### THE ROLE OF XCHK1 IN THE INHIBITION OF APOPTOSIS

Ayesha D. Carter and Jill C. Sible
Department of Biology, Virginia Tech, Blacksburg, VA 24061-0406 USA
aycarter@vt.edu

**INTRODUCTION.** Embryonic cell cycles of *Xenopus laevis* provide rare examples of non-pathological cell divisions that lack cell cycle checkpoints (1,2). Following fertilization, the *Xenopus* egg begins twelve rapid oscillations between DNA replication and mitosis without growth, gap phases, or checkpoints. Completion of the twelfth cleavage marks the midblastula transition (MBT) when transcription begins, and the cell cycle remodels, lengthening as it acquires gap phases, checkpoints and a program of apoptosis (3). We are investigating the relationship between *Xenopus* Chk1 (XChk1) signaling and apoptosis in the developing embryo.

**METHOD.** One-cell embryos were injected with mRNA encoding catalytically inactive XChk1 to inhibit endogenous XChk1 or luciferase as a control. To induce a DNA replication checkpoint, embryos were treated after the MBT with 100 μg/ml aphidicolin. DNA fragmentation associated with apoptosis was determined by whole mount TUNEL assays and by the resolution of DNA ladders from extracts of genomic DNA (1).

**RESULTS.** When treated with aphidicolin at Stage 9 of development (post-MBT), the embryos expressing inactive, dominant negative XChk1 exhibited a dramatic apoptotic-like cell death during gastrulation (Fig. 1). Subsequent resolution of genomic DNA from these embryos showed the DNA ladders characteristic of apoptotic death. Further analysis by TUNEL assay labeling the fragmented DNA confirmed that this death was due to apoptosis (Fig. 2).

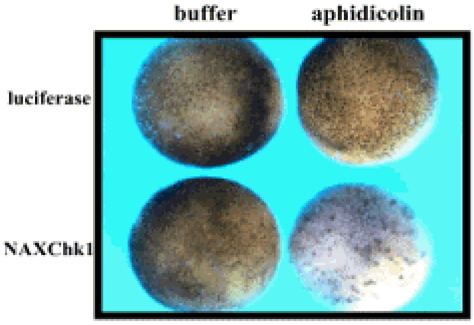

**Fig. 1 Inhibition of XChk1 induces a rapid death in embryos challenged with aphidicolin.**

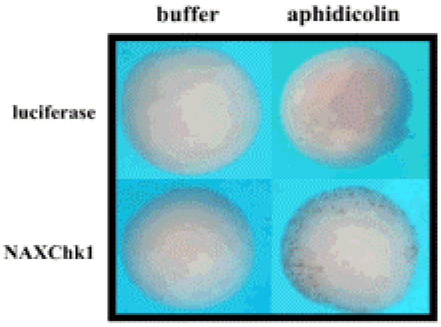

**Fig. 2 TUNEL assays confirm that this death is apoptotic.**

**DISCUSSION.** These experiments support the hypothesis that XChk1 normally blocks apoptosis after the MBT. We have recently determined that XChk1 is an inhibitor of Cdc2 and Cdk2 (4) and it is possible that this is the mechanism by which XCk1 inhibits apoptosis.

Therefore, XChk1 may function as a molecular switch to determine whether a cell will enter a cell cycle checkpoint (XChk1 on, Cdk2 off) or a program of apoptosis (XChk1 off, Cdk2 on).

**ACKNOWLEDGEMENTS.** This research was supported by grants from the National Institutes of Health (GM59688-01A1) to J. Sible, and by the State Dean Minority Fellowship to A. Carter.

**REFERENCES.**

1. Sible, J., Anderson, J., Lewellyn, A., and Maller, J. (1997) Dev. Biol. 189, 335
2. Anderson, J., Lewellyn, A., and Maller, J. (1997) Mol. Biol. Cell 8, 1195
3. Stack, J. and Newport, J. (1997) Development 124, 3185-3196
4. Kappas, N., Savage, P., Chen, K., Walls, A., and Sible, J. (2000) Mol. Biol. Cell 11, 3101-3108
